# Blocking KCa3.1 Channels Increases Tumor Cell Killing by a Subpopulation of Human Natural Killer Lymphocytes

**DOI:** 10.1371/journal.pone.0076740

**Published:** 2013-10-11

**Authors:** Shyny Koshy, Danli Wu, Xueyou Hu, Rajeev B. Tajhya, Redwan Huq, Fatima S. Khan, Michael W. Pennington, Heike Wulff, Patricia Yotnda, Christine Beeton

**Affiliations:** 1 Department of Molecular Physiology and Biophysics, Baylor College of Medicine, Houston, Texas, United States of America; 2 Center for Cell and Gene Therapy, Baylor College of Medicine, Houston, Texas, United States of America; 3 Graduate Program in Molecular Physiology and Biophysics, Baylor College of Medicine, Houston, Texas, United States of America; 4 Peptides International, Louisville, Kentucky, United States of America; 5 Department of Pharmacology, University of California, Davis, California, United States of America; Karolinska Institutet, Sweden

## Abstract

Natural killer (NK) cells are large granular lymphocytes that participate in both innate and adaptive immune responses against tumors and pathogens. They are also involved in other conditions, including organ rejection, graft-versus-host disease, recurrent spontaneous abortions, and autoimmune diseases such as multiple sclerosis. We demonstrate that human NK cells express the potassium channels Kv1.3 and KCa3.1. Expression of these channels does not vary with expression levels of maturation markers but varies between adherent and non-adherent NK cell subpopulations. Upon activation by mitogens or tumor cells, adherent NK (A-NK) cells preferentially up-regulate KCa3.1 and non-adherent (NA-NK) cells preferentially up-regulate Kv1.3. Consistent with this different phenotype, A-NK and NA-NK do not display the same sensitivity to the selective KCa3.1 blockers TRAM-34 and NS6180 and to the selective Kv1.3 blockers ShK-186 and PAP-1 in functional assays. Kv1.3 block inhibits the proliferation and degranulation of NA-NK cells with minimal effects on A-NK cells. In contrast, blocking KCa3.1 increases the degranulation and cytotoxicity of A-NK cells, but not of NA-NK cells. TRAM-34, however, does not affect their ability to form conjugates with target tumor cells, to migrate, or to express chemokine receptors. TRAM-34 and NS6180 also increase the proliferation of both A-NK and NA-NK cells. This results in a TRAM-34-induced increased ability of A-NK cells to reduce *in vivo* tumor growth. Taken together, our results suggest that targeting KCa3.1 on NK cells with selective blockers may be beneficial in cancer immunotherapy.

## Introduction

Natural killer (NK) cells are large granular lymphocytes that participate in both innate and adaptive immune responses, including the killing of cancerous cells [1], [2]. The ability to precisely regulate the activation and cytotoxicity of NK cell subsets is important in cancer immunotherapy.

Two potassium channels have been targeted for selective modulation of the function of subpopulations of T and B lymphocytes. These channels are the voltage-gated Kv1.3 (*KCNA3*) and the Ca^2+^-activated KCa3.1 channels (IKCa1, *KCNN4*) [3]–[5]. Their expression levels in human T lymphocytes depend upon the cells’ state of activation and differentiation [3]–[6]. At rest, all T lymphocytes express low levels of both channels. Following activation with a mitogen or an antigen, however, CCR7^+^ naïve and central-memory T cells up-regulate KCa3.1; In contrast, CCR7^−^ effector-memory T cells up-regulate Kv1.3. Similar to the switch observed in human T cells, the differentiation of human B lymphocytes from naïve and IgD^+^CD27^+^ B cells into class-switched memory B cells is accompanied by a switch from KCa3.1 to Kv1.3 [7]. Selective, potent, and safe blockers of both channels were generated [4], [5], [8]. In keeping with the differences in potassium channel expression levels, CCR7^−^ effector-memory T lymphocytes are preferentially sensitive to Kv1.3 blockers in proliferation, migration, and cytokine production assays whereas CCR7^+^ naïve and central-memory T cells depend on KCa3.1 for their function [4]–[6]. The difference in potassium channel expression is similarly responsible for a differential sensitivity of the B cell subsets to blockers of Kv1.3 or KCa3.1 [7].

In the mid-1980’s, voltage-gated (Kv) currents with properties resembling those of Kv1.3 were described in human NK cells and studies with non-selective Kv channel blockers demonstrated a functional role in NK cell cytotoxicity [9], [10]. Because the changes in potassium channel expression during T and B lymphocyte differentiation are important for the function of the respective T and B cell subsets, we examined the potassium channel phenotype of different human NK cell subpopulations. We show that human NK lymphocytes express both Kv1.3 and KCa3.1. While expression of these channels does not vary with expression levels of the classical maturation markers CD16 and CD56, it does vary between adherent and non-adherent NK cell populations as adherent NK (A-NK) cells preferentially up-regulate KCa3.1 and non-adherent (NA-NK) cells preferentially up-regulate Kv1.3. These phenotypic differences are accompanied by functional differences. The plasma membrane of A-NK cells is depolarized by TRAM-34, a selective KCa3.1 blocker, whereas the plasma membrane of NA-NK cells is depolarized by ShK-186, a selective Kv1.3 blocker. KCa3.1 blockade increases the proliferation, degranulation, and cytotoxicity of A-NK cells, but not of NA-NK cells. In contrast, a selective Kv1.3 blocker inhibits the proliferation and degranulation of NA-NK cells with minimal effects on A-NK cells.

## Materials and Methods

### Ethics Statement

The Institutional Review Board at Baylor College of Medicine determined that this work does not constitute human subjects research as buffy coats are de-identified and coded so the researchers have no means to trace a sample back to its donor.

All procedures using vertebrate animals were approved by the Institutional Animal Care and Use Committee at Baylor College of Medicine. Animals were euthanized by CO_2_ inhalation at the end of the experiments following guidelines to minimize suffering.

### Cells

The K562 erythroleukemia cell line was purchased from the ATCC (Manassas, VA). Buffy coats were purchased from the Gulf Coast Regional Blood Center (Houston, TX). NK cells were isolated by negative selection using the RosetteSep kit (StemCell Technologies, Vancouver, Canada) [11]. For further separation of A-NK and NA-NK cells, cells were incubated overnight in RPMI medium supplemented with 100 U/ml-100 µg/ml penicillin-streptomycin, 4 mM L-glutamine, 1 mM sodium pyruvate, 1% non-essential amino acids, 1% vitamins, and 5% FBS (all from Life Technologies, Grand Island, NY), 500 IU/ml rhIL-2, and 1 ng/ml rhIL-15 (R&D Systems, Minneapolis, MN). A-NK and NA-NK cells were collected, washed, and incubated for 24 hrs in cytokine-free medium.

### Potassium Channel Blockers

The selective KCa3.1 blockers TRAM-34 and NS6180 were synthesized and characterized for purity and activity on cells stably transfected with KCa3.1 channels as described [12], [13]. The selective Kv1.3 blockers ShK-186, ShK-192, and PAP-1 were synthesized and characterized for purity and activity on cells stably transfected with Kv1.3 channels as described [14]–[16]. Charybdotoxin, iberiotoxin, and apamin were purchased from CS Bio (Menlo Park, CA).

TRAM-34 blocks KCa3.1 channels with an IC_50_ of 20–25 nM and its IC_50_ on other KCa channels is above 10 µM (25 µM on KCa1.1, 18 µM on KCa2.1, 20 µM on KCa2.2, and 25 µM on KCa2.3) [12], [13]. NS6180 blocks KCa3.1 channels with an IC_50_ of 11 nM and displays IC_50_s above 10 µM on other KCa channels [13]. Both compounds were used at concentrations of 1 µM or below, in a concentration range in which both are selective for KCa3.1 channels.

### Patch-clamp Electrophysiology

All experiments were conducted in the whole-cell configuration of the patch-clamp technique with a holding potential of −80 mV using an EPC10-USB amplifier (HEKA Instruments, Bellmore, NY). Pipette resistances averaged 2.0 MΩ, and series resistance compensation of 80% was employed when currents exceeded 2 nA. Kv currents were recorded in normal Ringer solution containing (in mM): 160 NaCl, 4.5 KCl, 2 CaCl_2_, 1 MgCl_2_, 10 HEPES, pH 7.4, 290–310 mOsm, with a calcium-free pipette solution containing (in mM): 145 KF, 10 HEPES, 10 EGTA, 2 MgCl_2_, pH 7.4, 290–310 mOsm [6], [17]–[19]. Kv currents were elicited by repeated 200-ms depolarizing pulses from −80 mV to 40 mV, applied at intervals of 1 s to measure cumulative inactivation (“use-dependence”) or 30 s to allow time for Kv1.3 to go from the inactivated to the closed state between pulses [19]. Family of currents were elicited by 200-ms depolarizing pulses every 30 s from −60 mV to 60 mV in 10-mV increments. The half-activation voltage (V_1/2_) of the current was determined from the families of currents by plotting (G_K_/G_KMAX_) over V, where G_KMAX_ is determined at 40 mV [19]. IC_50_ values of Kv blockers were determined by fitting the Hill equation to the reduction of peak current measured at 40 mV during repeated pulses every 30 s [17]–[19]. For measurements of KCa currents we used an internal pipette solution containing (in mM): 145 K^+^ aspartate, 2 MgCl_2_, 10 HEPES, 10 K_2_EGTA and 8.5 CaCl_2_ (1 µM free Ca^2+^) or 2.28 mM CaCl_2_ (50 nM free Ca^2+^), pH 7.4, 290–310 mOsm. The external solution contained (in mM): 160 Na^+^ aspartate, 4.5 KCl, 2 CaCl_2_, 1 MgCl_2_, 5 HEPES, pH 7.4, 290–310 mOsm [12]. KCa currents were elicited by 200-ms voltage ramps from −120 mV to 40 mV applied every 30 sec and the reduction of slope conductance at −80 mV by the blockers was taken as a measure of channel block [12]. Kv1.3 and KCa3.1 numbers per cell were determined by dividing the whole-cell conductance by the single channel conductance value (12 pS for Kv1.3 and 11 pS for KCa3.1).

### [^3^H] Thymidine Incorporation

NK cells were plated into U-bottom 96-well plates (50,000 cells/well) in 100 µl culture medium. Kv1.3 and KCa3.1 blockers were diluted in culture medium and added to the appropriate wells (50 µl/well). Cells were incubated for 30–45 min at 37°C and then stimulated with 40 ng/ml PMA+500 nM ionomycin, or 500 IU/ml rhIL-2+1 ng/ml rhIL-15+PMA+ionomycin, all diluted in culture medium (50 µl/well). Cells were returned to the incubator for a total of 72 hrs. K562 cells were plated into flat-bottom 96-well plates (10,000 cells/well) in 100 µl culture medium. Kv1.3 and KCa3.1 blockers were diluted in culture medium and added to the appropriate wells (100 µl/well). Cells were incubated for a total of 72 hrs at 37°C. For both cell types, [^3^H] thymidine (1 µCi/well) was added during the last 16–18 hrs of the 72-hr culture. Plates were then frozen at −20°C to break the cells’ membranes and thawed before harvesting of DNA on glass fiber filters using a cell harvester (Inotech Biosystems International, Rockville, MD) and addition of scintillation liquid for counting of incorporated [^3^H] thymidine in a β scintillation counter (Beckman Coulter, Brea, CA) [14], [17], [18], [20]. In the control conditions without blockers, NK cells from different buffy coats proliferated at different rates. We therefore normalized the [^3^H] thymidine counts to proliferation levels obtained with the various stimuli in the absence of potassium channel blockers to eliminate this donor-induced variation.

### Degranulation Assays

NK cells were pre-incubated with channel blockers for 30–45 min before addition of K562 cells at a 1∶1 ratio. Degranulation of NK cells was measured 4 hours later by flow cytometry through exposure of CD107a to the plasma membrane of the NK cells, as described [21]–[23]. Data were acquired on a BD Canto II flow cytometer (BD Biosciences, San Jose, CA) with FACSDiva (BD Biosciences) and analyzed with FlowJo (Treestar, Ashland, OR) softwares. Data were normalized to the results obtained in the presence of K562 cells without potassium channel blockers to eliminate donor-induced variation.

### Cytotoxicity Assays

K562 cells were labeled with 0.5 µM carboxyfluorescein diacetate, succinimidyl ester (CFSE; Life Technologies, Grand Island, NY). NK cells were pre-incubated with 1 µM TRAM-34 or 100 nM ShK-186 and mixed with labeled K562 cells at various NK:K562 ratios for 4 hours at 37°C. Dead cells were stained with 7-AAD (Sigma-Aldrich, St Louis, MO) as described [24] and data acquired by flow cytometry on a BD Canto II. For each buffy coat, data were normalized to the baseline killing of K562 cells in the absence of potassium channel blockers at the different NK:K562 ratio to eliminate donor variation.

### Membrane Potential Measurements

The membrane potential of A-NK and NA-NK cells was measured by flow cytometry using DiBAC_4_(3) (Life Technologies). Cells were washed and resuspended in PBS. They were incubated for 10 min with either 100 nM ShK-186 or 1 µM TRAM-34 before addition of 150 nM DiBAC_4_(3). This concentration of dye was chosen after running an assay with varying concentrations of the dye (10–2000 nM). In each case, dye equilibration was allowed for 2 min at room temperature, after which fluorescence was measured on a Canto II flow cytometer (BD Biosciences).

### Conjugate Formation

NK cells were labeled with CFSE and K562 cells were labeled with PKH26 (Sigma-Aldrich) following manufacturers’ guidance. Stained cells were mixed at a ratio of 1∶1. Formation of conjugates was evaluated for each combination after a 20 min incubation at 37°C by quantifying the CFSE^+^PKH26^+^ double positive signal by flow cytometry, as described [25].

### Migration Assays

NK cell migration was assayed in 24-well plates using transwell chambers with a 5 µm pore membrane (Millipore, Billerica, MA) using a protocol adapted from [26]. Serum-starved A-NK or NA-NK cells were resuspended in serum-free medium and added to the inserts (2×10^5^/100 µl per insert). Control (serum-free) or serum-containing medium was added to the lower part of the wells. Plates were incubated at 37°C for 4 hours. Migrated cells in the lower chamber were counted using a hemocytometer.

### Detection of Cell Surface Markers by Flow Cytometry

NA-NK and A-NK cells were cultured overnight in the presence of 1 µM TRAM-34 or vehicle. Their surface phenotype was investigated using fluorophore-conjugated monoclonal antibodies specific for CCR1, CCR2, CXCR3, CD62L (R&D Systems), CCR5, CXCR4, CX3CR1 (eBioscience, San Diego, CA), CD16, CD56 (BD Biosciences). Corresponding isotype controls were systematically used to compensate for nonspecific signals. Cells were first incubated with Fc receptor binding inhibitor (eBioscience), stained with antibodies as recommended by the manufacturer, and analyzed using a FACSAria flow cytometer (BD Biosciences).

### Mice and Adoptive Transfers

NOD-SCID mice (6–8 weeks old) bred at Baylor College of Medicine were sublethally irradiated (3.5 Gy). The next day, they received a subcutaneous injection of 1 million K562 cells in Matrigel in their flank [27]. After tumor engraftment, mice with similar tumor sizes were divided into 6 groups. Mice in negative control groups received the vehicle Miglyol-812 (caprylic/capric triglyceride; Spectrum chemicals, New Brunswick, NJ) or the blocker (TRAM-34 in vehicle; 120 mg/kg subcutaneous in the scruff of the neck daily). Animals in positive control groups received human A-NK or NA-NK cells (5 million/mouse intravenously), but no blocker, and those in test groups received TRAM-34 and either A-NK or NA-NK cells. All mice received rhIL-15 and rhIL-2 to support NK cell proliferation and survival *in vivo* [28]. Tumor size was measured with a caliper. Animals were euthanized by CO_2_ inhalation at the end of the experiments following guidelines to minimize suffering.

### Statistical Analysis

We used the non-parametric Mann-Whitney *U*-test to calculate statistical significance of all of our results (GraphPad Prism Software). *P* values less than 0.05 were considered significant.

## Results

### Identification of Kv1.3 and KCa3.1 in NK Cells

**Figure 1:**
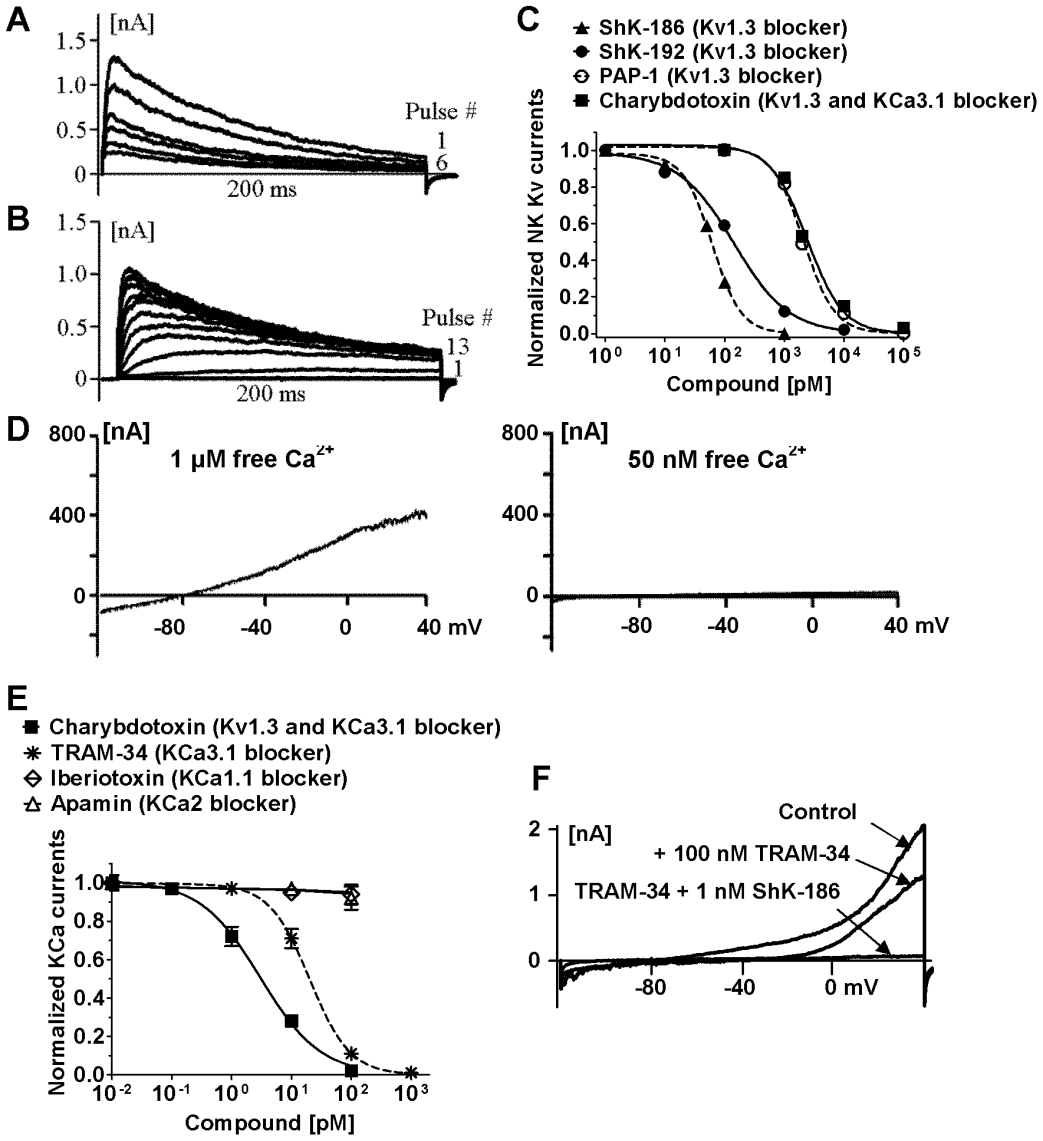
Human NK cells express functional Kv1.3 and KCa3.1. **A:** Cumulative inactivation of Kv currents. Cells were pulsed to 40−80 mV every second for 200 ms. **B:** Family of Kv currents. The test potential was changed from −60 to 60 mV in 10-mV increments every 30 s. **C:** Dose-dependent inhibition of Kv currents by ShK-186 (▴; IC_50_∶ 61±3 pM), ShK-192 (•; IC_50_∶ 142±22 pM), PAP-1 (○; IC_50_∶ 2.1±0.2 nM), and charybdotoxin (▪; IC_50_∶ 2.4±0.4 nM). **D:** KCa currents during 200-ms ramp pulses with an internal solution containing 1 µM or 50 nM free Ca^2+^. **E:** Dose-dependent inhibition of KCa currents by charybdotoxin (▪; IC_50_∶ 3±0.4 nM), TRAM-34 (*; IC_50_∶ 20±0.4 nM), iberiotoxin (◊), and apamin (Δ). **F:** Complete block of KCa and Kv currents by a combination of TRAM-34 and ShK-186.

We isolated human NK cells (93–98% CD3^−^CD56^+^ by flow cytometry) and used established whole-cell patch-clamp protocols to identify the potassium channels expressed at their plasma membrane without further stimulation or separation. Patch-clamp electrophysiology is the “gold-standard” technique to detect, identify, and quantify functional ion channels in cell membranes [29]. Most cells (92±8%) exhibited a Kv current with the biophysical and pharmacological fingerprint of cloned Kv1.3 and of Kv1.3 described in T and B lymphocytes [6], [7], [12], [19]. Pulsing the cells to 40 mV for 200 ms induced an outward potassium current through fast opening and slowly inactivating Kv channels (Fig. 1A, pulse # 1). Rapid pulsing every second reduced current amplitude at every pulse in a “use-dependent” manner, a characteristic property of the Kv1.3 channel, which needs 30 sec to go from the inactivated to the closed conformation following 200 ms pulses (Fig. 1A). Pulsing the cells to −60 mV was not sufficient to induce Kv channel opening (Fig. 1B, pulse # 1). Increase in the voltage applied at every pulse by 10 mV every 30 sec induced increasing current amplitudes, showing that the current is voltage-gated (Fig. 1B). The voltage sufficient to open half of the Kv channels (V_1/2_) was −32±0.5 mV, the value previously described for Kv1.3. The blockers ShK-186, ShK-192, PAP-1, and charybdotoxin blocked Kv currents with IC_50_s similar to those previously described for homotetramers of cloned and native Kv1.3 in T lymphocytes [4], [5], [8], [12], [16] (Fig. 1C). These data indicate that the functional Kv channel at the plasma membrane of human NK cells is Kv1.3.

A small number of NK cells (6±4%) expressed a calcium-activated potassium (KCa) channel but no Kv channel as demonstrated by linear currents only in the presence of sufficient concentrations of free cytoplasmic calcium (Fig. 1D). The KCa family of channels includes several members, we therefore used known blockers of the different KCa channels in whole-cell patch-clamp assays to further identify the KCa channel expressed by NK cells. The KCa channel was insensitive to the KCa1.1 channel blocker iberiotoxin at concentrations known to block >90% of this channel [30] and to the KCa2.1, KCa2.2, and KCa2.3 channel blocker apamin at concentrations known to block >90% of these channels [31] (Fig. 1E), indicating that neither of these channels underlie the KCa current. The KCa current was however sensitive to both TRAM-34 and charybdotoxin with IC_50_s in the low nanomolar range, consistent with published IC_50_ values for these blockers on cloned and native KCa3.1 in T lymphocytes [8], [12], [13].

The majority (78±8%) of cells displayed both a KCa and a Kv current as evidenced by linear potassium currents at low voltages and curving upwards at higher voltages (Fig. 1F, control pulse). The addition of TRAM-34 inhibited the KCa component of the current, leaving only the Kv component visible at higher voltages. A combination of TRAM-34 and ShK-186 completely suppressed the potassium currents, suggesting KCa3.1 and Kv1.3 are the only functional KCa and Kv channels at the plasma membrane of human NK cells (Fig. 1F).

### The Kv1.3 and KCa3.1 Phenotype of NK Cells Varies with Their Adhesive Properties Following Incubation with rhIL-2 and rhIL-15

**Figure 2:**
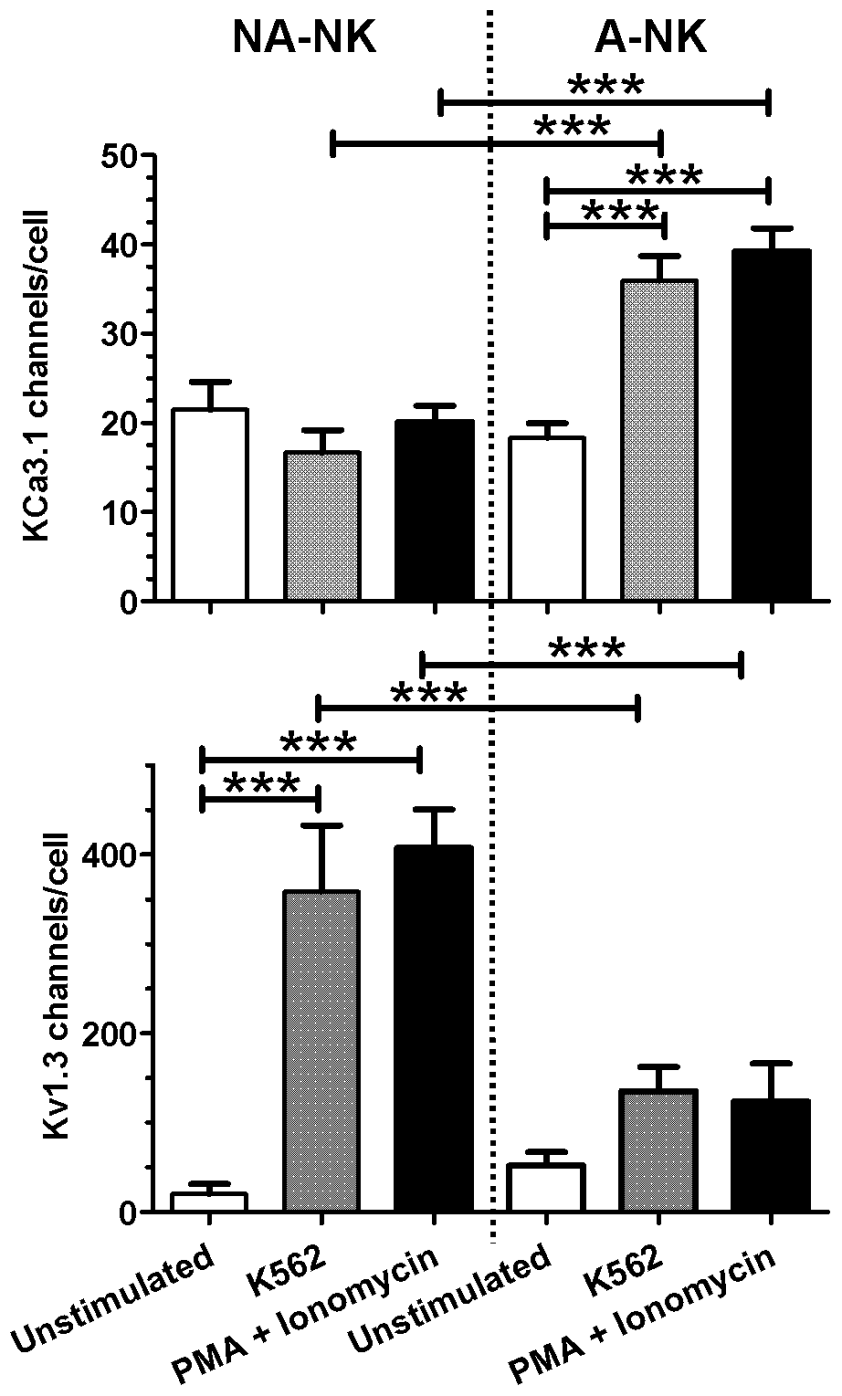
Activation induces an up-regulation of Kv1.3 by NA-NK cells and of KCa3.1 by A-NK cells. Cells were patch-clamped in the whole-cell configuration without stimulation and after stimulation with either an equal number of K562 cells or 40 ng/ml PMA +500 nM ionomycin for 24 hours and numbers of KCa3.1 (*top*) and Kv1.3 (*bottom*) channels were calculated. Each bar represents the mean ± SEM channel numbers. Cells from a minimum of 3 donors were used for each condition and a minimum of 10 cells were analyzed for each donor.

Since we have found that human peripheral blood NK cells are heterogeneous in terms of KCa3.1 and Kv1.3 expression, we separated the cells based on CD16 and CD56 expression levels and assessed their potassium channel phenotype. We used whole-cell patch-clamp electrophysiology to identify the channels. All subsets (CD16^+^CD56^dim^, CD16^−^CD56^dim^, CD16^−^CD56^bright^) expressed a combination of Kv1.3 and KCa3.1 at various ratios without any visible trend (Table 1). Similar diversity was observed when separating the cells based on expression levels of CD62L (Table 1). We next separated the NK cells based on their ability to adhere to plastic following overnight incubation with rhIL-2 and rhIL-15 [32]–[35]. The majority of the CD3^−^CD56^+^ cells remained non-adherent (NA-NK) but 11±2% adhered to plastic (A-NK). The adhesive properties of NK cells did not correlate with expression levels of CD16, CD56, CD11b, CD27, CD62L, CCR7, CD134, CD90 (Fig. S1). Both A-NK and NA-NK expressed the activation marker CD69 but its expression level was higher in A-NK than NA-NK cells (*p*<0.05) (Fig. S1). Whole-cell patch-clamp analysis of A-NK and NA-NK cells 24 hours after separation and culture in cytokine-free medium revealed a low expression of both KCa3.1 and Kv1.3 (Fig. 2). After determining the whole-cell potassium conductance, we then calculated the number of Kv1.3 and KCa3.1 channels in each cell based on their respective single channel conductances. NA-NK cells expressed 20±11 Kv1.3/cell and 21±3 KCa3.1/cell while A-NK cells expressed 52±15 Kv1.3/cell and 18±2 KCa3.1/cell. Following incubation for 24 hrs with K562 cells or with PMA and ionomycin, KCa3.1 numbers remained unchanged in NA-NK cells but increased in A-NK cells to 36±3 channels/cell (K562) and 39±3 channels/cell (PMA+ionomycin). In contrast, NA-NK, but not A-NK cells, up-regulated Kv1.3 to 358±74 channels/cell (K562) and 407±43 channels/cell (PMA+ionomycin).

**Table 1:** Percentages of CD3^−^CD56^+^ NK cells that express Kv1.3, KCa3.1, or both channels after cell-sorting of different populations based on cell surface marker expression.

NK cell subset	% that express both Kv1.3 and KCa3.1	% that express mainly Kv1.3	% that express mainly KCa3.1
CD16^−^CD56^dim^	76±3.9, n = 10	17±9.3, n = 9	7±4.6, n = 6
CD16^−^CD56^bright^	75±8.1, n = 7	18±3.0, n = 6	7±2.1, = 6
CD16^+^CD56^dim^	79±8.1, n = 8	14±5.3, n = 15	7±5.1, n = 8
CD62L^hi^	83±8.1, n = 15	15±9.4, n = 13	2±1.7, n = 8
CD62L^−/lo^	80±7.8, n = 15	16±4.6, n = 14	4±2.7, n = 8

Cells from 6 donors were patch-clamped in the whole-cell configuration (3 after separation based on expression of CD16 and CD56 and 3 after separation based on expression of CD62L). Data are given as mean ± SEM with n = number of cells patch-clamped in each population.

### KCa3.1 Block Increases the Proliferation of Both NK Cell Subsets whereas Kv1.3 Block Inhibits the Proliferation of NA-NK, but not of A-NK Cells

**Figure 3:**
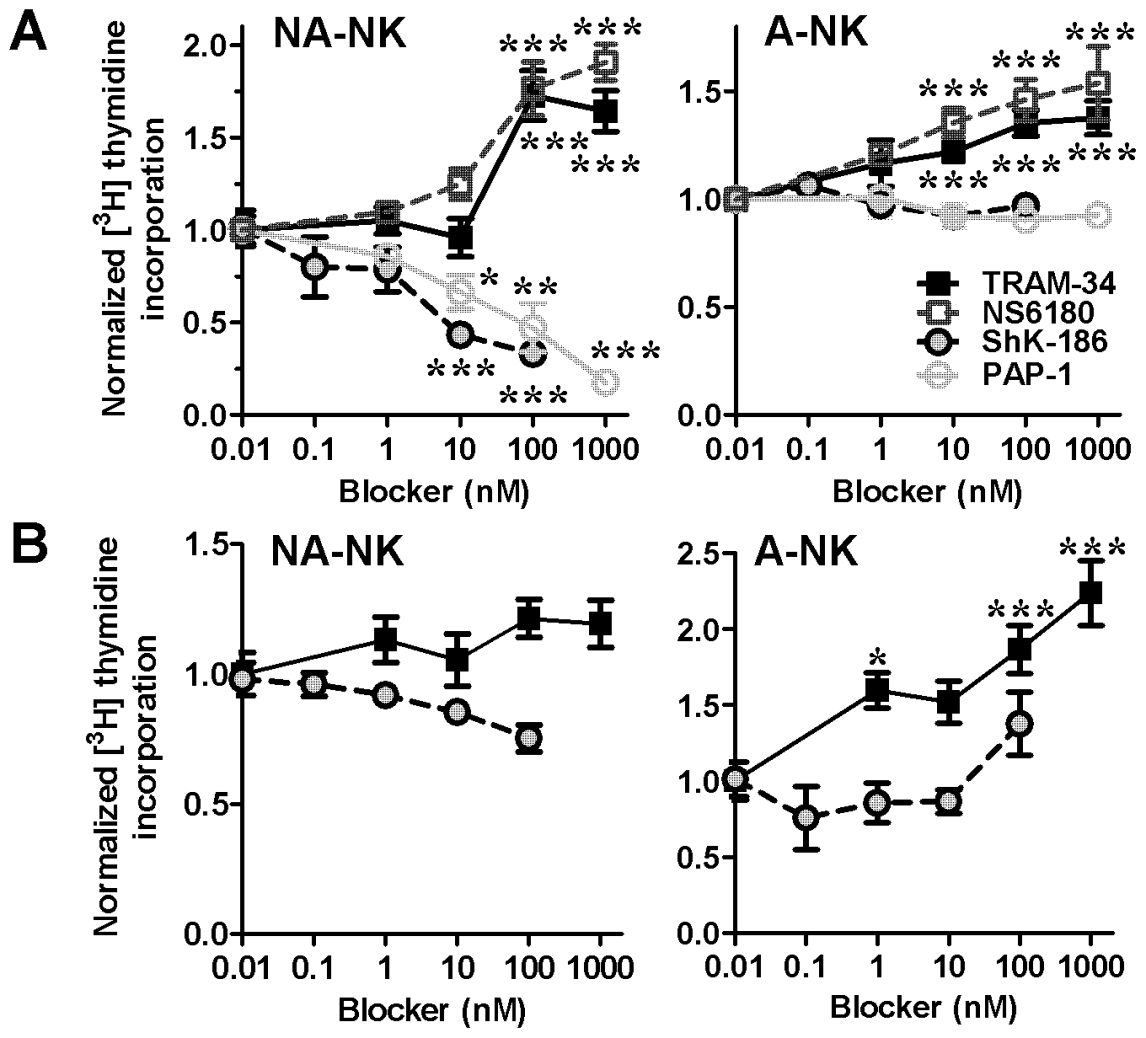
Blocking KCa3.1 and Kv1.3 differentially affects the proliferation of A-NK and NA-NK cells. Dose-dependent effects of TRAM-34 (▪; n = 7 donors each analyzed in triplicates), NS6180 (□; n = 3 donors each analyzed in triplicates), ShK-186 (•; n = 7 donors each analyzed in triplicates), and PAP-1 (○; n = 7 donors each analyzed in triplicates) on [^3^H] thymidine incorporation by NA-NK and A-NK stimulated with PMA and ionomycin alone (**A**) or in addition to rhIL-2 and rhIL-15 (**B**). Data represent the mean ± SEM. *p<0.05, **p<0.01, ***p<0.001.

Since A-NK and NA-NK express different Kv1.3 and KCa3.1 numbers following activation, we assessed the effects of blocking these channels on NK cell proliferation. Blocking Kv1.3 with ShK-186 or PAP-1 resulted in significant reduction of NA-NK cell proliferation (IC_50_ ≈ 7 nM for ShK-186 and ≈ 50 nM for PAP-1) but did not affect the proliferation of A-NK cells (Fig. 3A). In contrast, blocking KCa3.1 with TRAM-34 or NS6180 enhanced the proliferation of both NA-NK and A-NK cells.

Addition of rhIL-2 and rhIL-15 during the mitogenic stimulation abolished the effects of ShK-186 on the proliferation of both NA-NK and A-NK cells (Fig. 3B), as was previously reported with IL-2 in T lymphocytes [20], [36]. The enhancing effects of TRAM-34 on the proliferation of A-NK cells were unaffected by rhIL-2 and rhIL-15 but those on NA-NK cells were significantly reduced.

### KCa3.1 Blockade Increases the Degranulation of A-NK Cells and their Ability to Kill K562 Cells

One of the major functions of NK cells is to release cytotoxic granules to kill target cells. We assessed the effects of Kv1.3 and KCa3.1 blockers on the ability of A-NK and NA-NK cells to degranulate in the presence of K562 cells and to kill these tumor cells.

**Figure 4:**
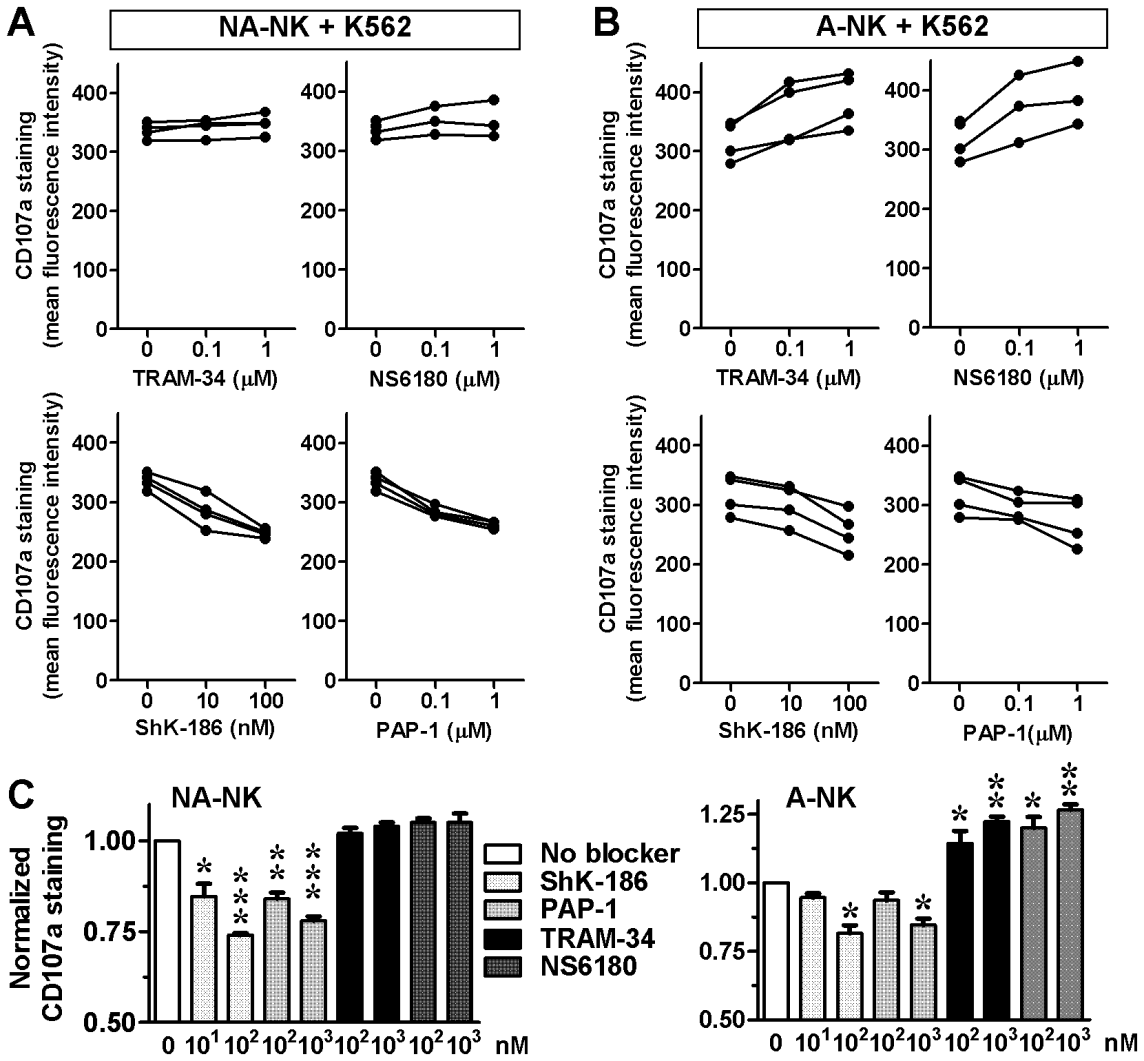
Blocking KCa3.1 and Kv1.3 differentially affects the degranulation of A-NK and NA-NK cells. **A:** Mean fluorescent intensity of CD107a staining in NA-NK and A-NK cells in the presence of the concentrations of blockers indicated on the X-axis of each plot. Each data point represents the mean of duplicate assays on single donors. **B:** CD107a expression levels in NA-NK and A-NK cells in the presence of K562 cells without potassium channel blockers (white) or in the presence of ShK-186 (10 and 100 nM; light grey), PAP-1 (0.1 and 1 µM; medium grey), TRAM-34 (0.1 and 1 µM; black), or NS6180 (0.1 and 1 µM; dark grey). Data represent the mean ± SEM of data obtained in triplicate in independent assays with cells from the donors shown in panel A. *p<0.05, **p<0.01, ***p<0.001.

Both ShK-186 and PAP-1 significantly inhibited the degranulation of NA-NK cells (Fig. 4A, C). The Kv1.3 blockers also inhibited the degranulation of A-NK cells, but only when used at high concentrations (Fig. 4B, C). Blocking KCa3.1 with TRAM-34 or NS6180 did not affect the degranulation of NA-NK cells (Fig. 4A, C) but significantly enhanced the degranulation of A-NK cells (Fig. 4B, C). In the absence of K562 cells, none of the blockers induced degranulation of NK cells (data not shown).

**Figure 5:**
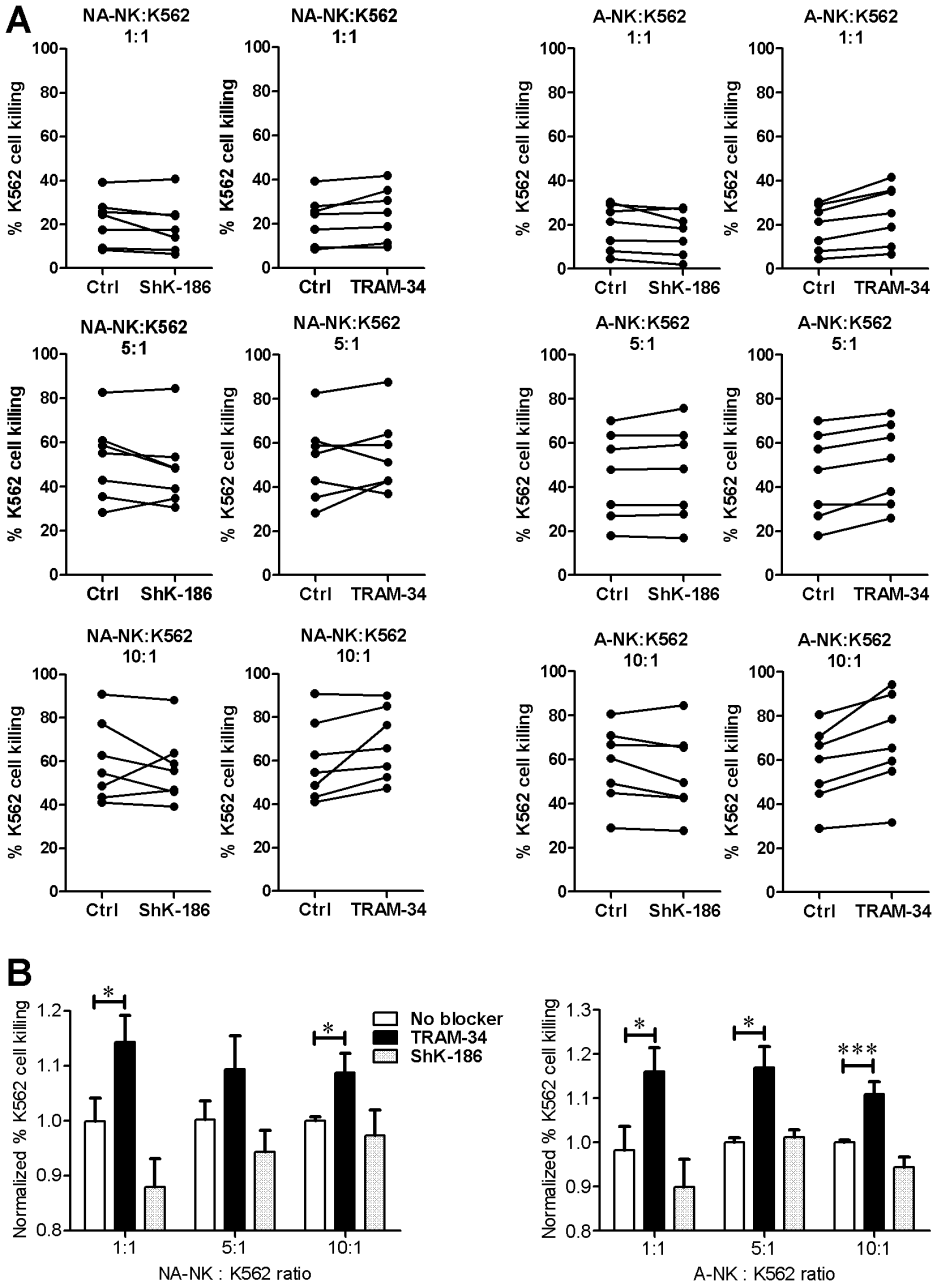
Blocking KCa3.1 and Kv1.3 differentially affects the cytotoxicity of A-NK and NA-NK cells. **A:** Percentage of dead target K562 cells after incubation with NA-NK or A-NK cells at different NK:K562 ratio in the absence of blocker (left of each plot) or in the presence of 100 nM ShK-186 or 1 µM TRAM-34 (right of each plot). Each data point represents the mean of duplicate assays on single donors. **B:** Data shown in A normalized for each of the 7 donors to the mean value obtained in the absence of potassium channel block. White, no blocker; grey, 100 nM ShK-186; black, 1 µM TRAM-34. Data represent the mean ± SEM of data obtained in duplicate with cells from 7 donors in 7 independent experiments. *p<0.05, **p<0.01, ***p<0.001.

KCa3.1 blockade did not induce an increase in the number of degranulating cells but rather an increase in the level of degranulation of A-NK cells in the presence of K562 cells. We therefore used 7-AAD staining to determine whether the TRAM-34-induced increase in degranulation results in increased killing of K562 target cells. TRAM-34 significantly increased the cytotoxicity of A-NK cells against K562 cells at all effector:target ratios tested by 11.2±0.3%, 11.0±1.0%, and 11.6±0.6% (ratio of 10∶1, 5∶1, and 1∶1, respectively) (Fig. 5). TRAM-34 increased the killing of K562 cells by NA-NK. ShK-186 did not significantly affect the cytotoxicity of either A-NK or NA-NK against K562 cells.

### TRAM-34 Induces a Depolarization of the Plasma Membrane of A-NK Cells and ShK-186 Depolarizes that of NA-NK Cells

**Figure 6:**
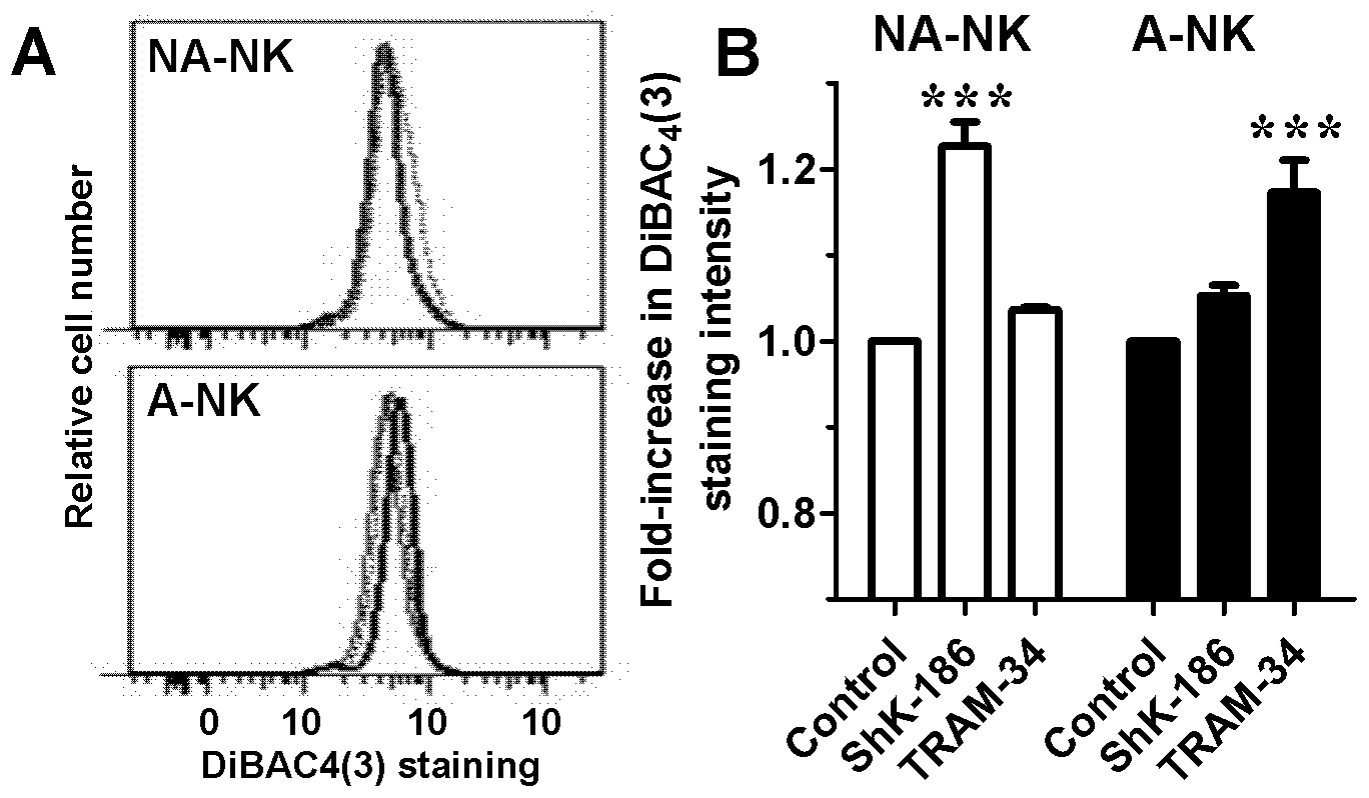
Blocking KCa3.1 and Kv1.3 differentially affects the membrane potential of A-NK and NA-NK cells. **A:** Representative flow cytometric histograms comparing DiBAC_4_(3)-stained NA-NK (top) and A-NK (bottom) cells treated with 100 nM ShK-186 (dotted lines), 1 µM TRAM-34 (thick lines) or vehicle (thin lines). **B:** Effects of 1 µM TRAM-34 and 100 nM ShK-186 on DiBAC_4_(3) staining intensity in NA-NK (white) and A-NK (black) cells. Data represent the mean ± SEM of data obtained in duplicate with cells from 5 donors.

Potassium channels regulate the membrane potential of T lymphocytes [37], [38]. To determine if they play a similar role in NK lymphocytes, we tested the effects of potassium channel blockers on NK cell membrane potential. TRAM-34 induced a depolarization of the plasma membrane of A-NK cells but not of NA-NK cells (Fig. 6). In contrast, ShK-186 induced a depolarization of the plasma membrane of NA-NK cells with only minimal effects on the membrane potential of A-NK cells.

### TRAM-34 does not Affect the Conjugation of NK Cells with K562 Cells

**Figure 7:**
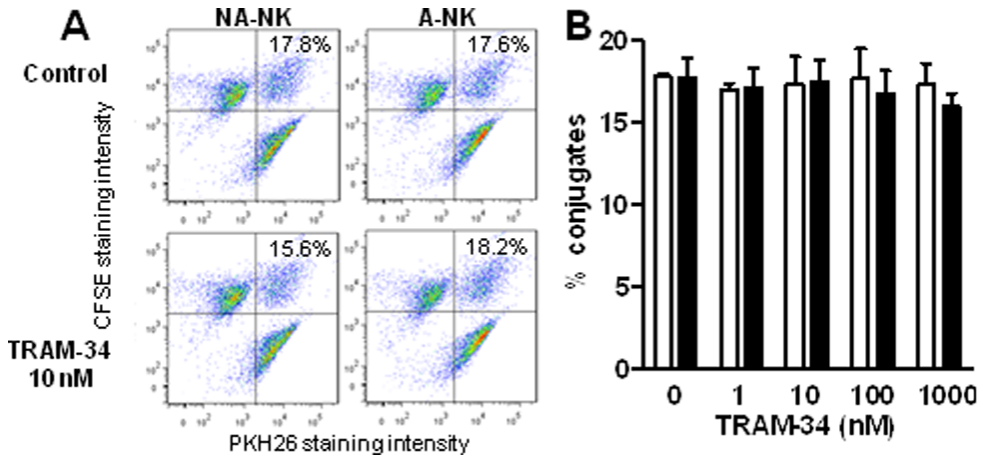
TRAM-34 does not affect the formation of conjugates between NK and K562 cells. **A:** Representative dot-plots with double positive signals representing the conjugates formed by NK and K562 cells, the percent of conjugates are indicated in each condition. **B:** Percent of NK:K562 conjugates obtained in absence or presence of 1 µM TRAM-34 for A-NK (black) and NA-NK (white). Data represent the mean ± SEM of data obtained with cells from 6 donors. *p<0.05, **p<0.01, ***p<0.001.

To assess whether the increased degranulation of A-NK cells in response to K562 cells was accompanied by a better formation of A-NK:K562 cells conjugates, we measured conjugate formation by flow cytometry. TRAM-34-treated A-NK cells produced a similar number of conjugates with K562 cells as non-treated A-NK cells (Fig. 7). Similar results were obtained with NA-NK cells.

### TRAM-34 does not Affect the NK Cell Migration or Expression of most Chemokine Receptors

**Figure 8:**
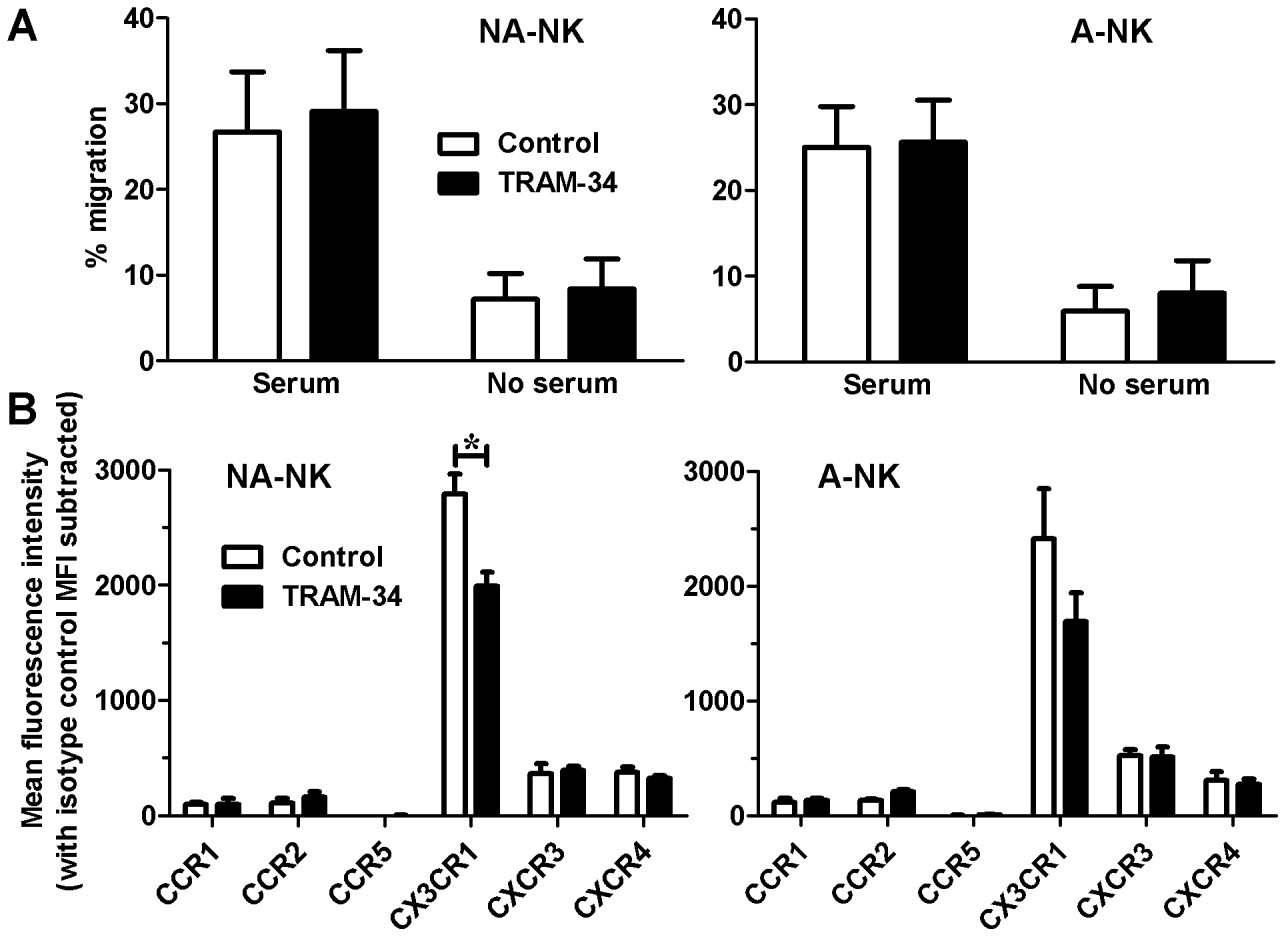
Blocking KCa3.1 affects the expression levels of CX3CR1 but not of other chemokine receptors or NK cell migration. **A:** Effect of 1 µM TRAM-34 (black) and vehicle (white) on the migration of NA-NK (left) and A-NK (right) cells in the presence and absence of serum. Data represent the mean ± SEM of data obtained with cells from 6 donors. **B:** Effect of 1 µM TRAM-34 (black) and vehicle (right) on the expression levels of chemokine receptors by NA-NK (left) and A-NK (right) cells. Data represent the mean fluorescence intensity with anti-chemokine receptor antibodies after subtracting the background mean fluorescence intensity in the presence of isotype control. N = 3 donors. *p<0.05.

To investigate the effect of blocking KCa3.1 on their migration, A-NK and NA-NK cells, either untreated or treated with 1 µM TRAM-34, were used in transwell assays. We observed minimal (<10%) cell migration in control wells (no serum) (Fig. 8A). Serum induced the migration of both subsets of NK cells and neither was affected by TRAM-34.

Since homing of NK cells requires expression of chemokine receptors, we used flow cytometry to determine the effects of blocking KCa3.1 channels with TRAM-34 on the expression of CCR1, CCR2, CCR5, CX3CR1, CXCR3, and CXCR4. TRAM-34 reduced the expression of CX3CR1 on NA-NK cells (Fig. 8B) but did not affect the expression of the other chemokine receptors in either NA-NK or A-NK cells.

### TRAM-34 Increases the Potency of Adherent NK Cells to Control K562 Tumor Growth in Mice

**Figure 9:**
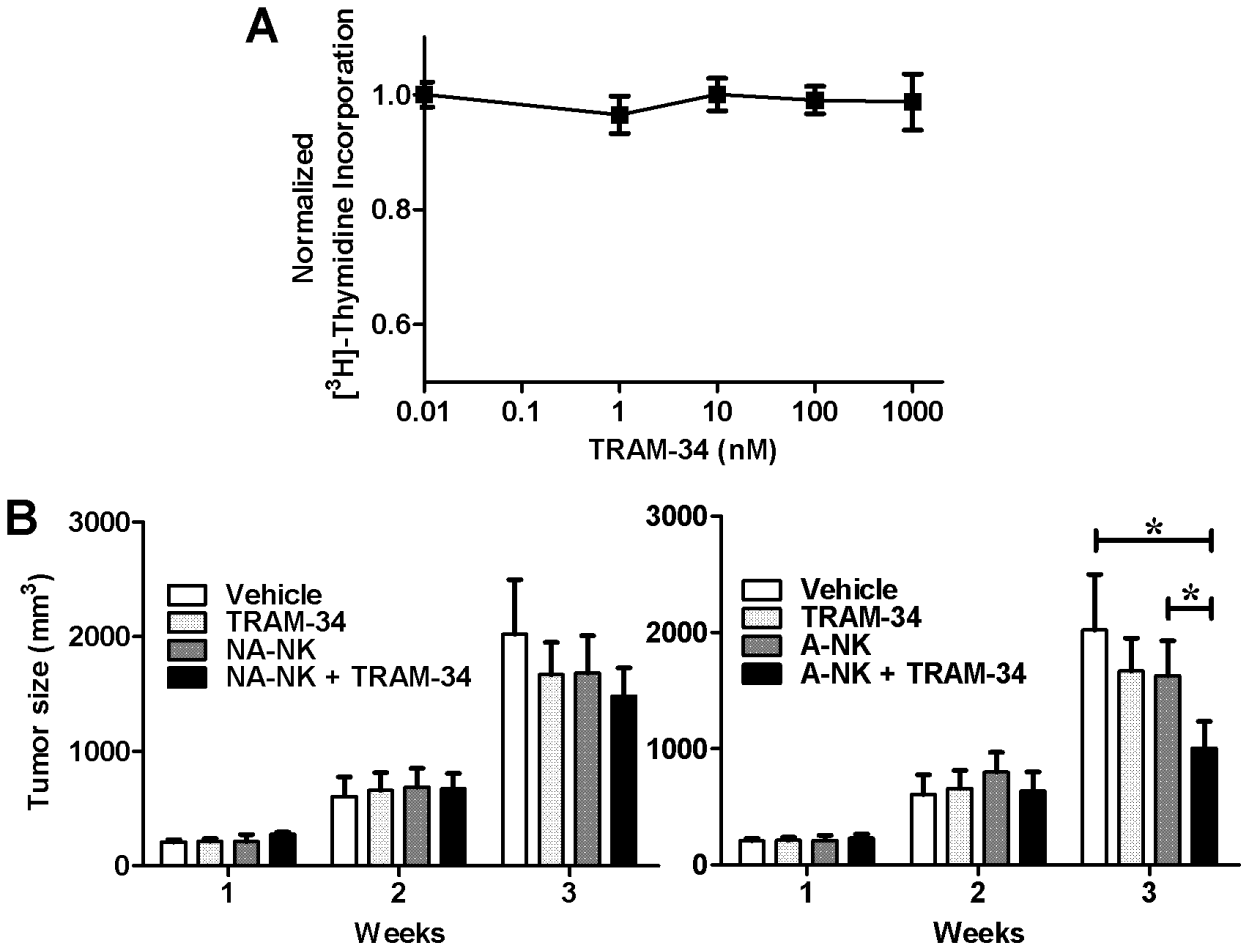
TRAM-34 promotes A-NK cell antitumor activity *in vivo*. **A:** Lack of effects of TRAM-34 on [^3^H] thymidine incorporation by K562 cells. N = 4 independent experiments. **B:** Effect of NA-NK (*left*) and NA-NK (*right*) cells on the size of K562 tumors in the absence (dark grey) and presence (black) of 120 mg/kg TRAM-34. Vehicle control in shown in white and TRAM-34 in the absence of NK cells in light grey. Data represent the mean ± SD tumor size for each group (n = 5 mice/group) at each time point. *p<0.05.

We used a xenograft mouse model to evaluate the *in vivo* effects of KCa3.1 blockade on both NK populations. We chose TRAM-34 for the *in vivo* experiments as it has a better bioavailability than NS6180 [13], [39]. As a first step, we established that tumors formed by K562 cells are not sensitive to KCa3.1 block by testing the effects of TRAM-34 on the proliferation of K562 cells *in vitro*. We found that it had no effect over a 72-hour period at concentrations up to 1 µM (Fig. 9A). This confirmed that we could use K562 tumor cells for the *in vivo* experiments.

In order to remain close to physiological conditions and to limit bias induced by prolonged culture, we used fresh NK cells cultured only for the separation of A-NK and NA-NK cells. *In vivo* experiments were therefore limited by the number of A-NK cells available from each buffy coat. To ensure we compared mice reconstituted with cells from the same donor in the different treatment groups, we administered a single intravenous injection of 5 million A-NK or NA-NK cells per mouse. Mice that received the vehicle alone had the largest tumors (Fig. 9B). A-NK or NA-NK cells administered in the absence of TRAM-34 did not significantly reduce tumor growth; neither did treatment with TRAM-34 alone nor administration of NA-NK cells and TRAM-34. A significant tumor growth delay (50±11%, p<0.05) was only observed in mice that had received both A-NK cells and TRAM-34. These results suggest that TRAM-34 potentiates the antitumor activity of A-NK but not of NA-NK cells.

## Discussion

Human T and B lymphocytes express Kv1.3 and KCa3.1 and previous studies have shown the expression of a Kv channel resembling Kv1.3 by human NK cells [9], [10]. Here, we use whole-cell patch-clamp electrophysiology, the “gold-standard” technique for ion channel identification and quantification, to show that human NK cells express the same Kv and KCa channels as human T and B lymphocytes [6], [7] and that the expression levels of these channels vary between the different subsets of NK cells, as was shown for T and B cells. Using selective blockers, we furthermore show that this difference in Kv1.3 and KCa3.1 expression levels is important in regulating the membrane potential, degranulation and proliferation of the different subsets of NK cells, resulting in an increase in cytotoxicity against K562 target cells, both *in vitro* and *in vivo*. Blocking these channels did however not affect the ability of NK cells to migrate or form conjugates with K562 cells.

Potential off-target effects are always of concern when using pharmacological tools. We have therefore used two structurally unrelated KCa3.1 blockers, TRAM-34 [12] and NS6180 [13], in key assays of our study. Both blockers have been found selective for KCa3.1 when used at concentrations up to 1 µM [13], which is the maximum concentration used in all of our assays. The compounds yielded similar results, confirming that their effects on NK cells are caused specifically through KCa3.1 blockade. We similarly used ShK-186 [40] and PAP-1 [16] to ensure the observed effects are due to Kv1.3 block. ShK-186 and PAP-1 concentration of 70 pM and 2 nM are sufficient to block 50% of the Kv1.3 current of NK cells and concentrations of 20 nM of TRAM-34 and 11 nM of NS6180 are sufficient to block 50% of their KCa3.1 currents. However, concentrations in the nM or low µM are necessary to affect proliferation, degranulation, and cytotoxicity. Such a difference in blocker efficacy in whole-cell patch-clamp and functional assays was previously observed with these and other potassium channel blockers in other cell types, including T lymphocytes [6], [14], [15], [17], [41], [42]. At least three factors may influence this difference. First, a block of >90% of the channels may be necessary to alter cell function, requiring much higher concentrations of blockers to observe an effect on proliferation or degranulation. Second, the tissue culture medium used for functional assays contains serum and is more complex than the well-defined salt solutions used for patch-clamp; blockers may bind to medium components (such as FBS proteins), reducing the concentration of the free and active form of the blocker available for channel block (so called “serum-shift”). Finally, the patch-clamp results are a direct readout of the effect of the blockers on the channels but during functional assays, multiple pathways with different sensitivities to potassium channel block are likely activated and contribute to the final readout.

Previous studies demonstrated a role of Kv channels in regulating the ability of human NK cells to kill target K562 cells as non-selective blockers of Kv channels, including Kv1.3, inhibited cytotoxicity [9], [10]. Here, we show that selective block of Kv1.3 does indeed inhibit the degranulation of NA-NK cells in the presence of K562 cells but has limited effects on the degranulation of A-NK cells. Since A-NK cells represent only ∼10% of the CD3^−^CD56^+^ NK cell population, the lack of effect of Kv1.3 blockers on their degranulation and cytotoxicity could be masked by the inhibitory effect on the NA-NK cells that represent the majority of circulating NK cells when studying bulk NK cells. In A-NK cells, blocking KCa3.1 with TRAM-34 or NS6180 induced an increase in degranulation. This KCa3.1 blocker-induced increase in degranulation was paralleled by an increase in cytotoxicity but the ShK-186-induced decrease in degranulation by NA-NK cells was not. Non-selective Kv channel blockers have been shown to reduce cytotoxicity by human NK cells [9], [10]. In this previously published work, NK cells were used immediately after isolation whereas our current study required their overnight incubation with rhIL-2 and rhIL-15 to separate A-NK and NA-NK. The addition of IL-2 is known to reverse the inhibitory effects of Kv1.3 blockers on the proliferation of T lymphocytes [20], [36]. Although we used the NK cells after 24 hours in cytokine-free medium, their required contact with rhIL-2 and rhIL-15 for cell separation may have triggered signaling events resulting in a reduced efficacy of ShK-186 in inhibiting NK cell cytotoxicity, leading to this discrepancy between our and published data. Indeed, the addition of these cytokines abolished the effects of Kv1.3 block on the proliferation of both NA-NK and A-NK cells. In addition, 65–70% of both A-NK and NA-NK cells expressed the activation marker CD69 whereas only 15% of freshly-isolated NK cells did (Fig. S1). Although the majority of A-NK and NA-NK cells expressed CD69, expression levels assessed by mean fluorescence intensity were significantly higher in A-NK than in NA-NK cells. This suggests a stronger activation of the A-NK cells by incubation with IL-2 and IL-15 and may explain in part the differential expression of Kv1.3 and KCa3.1 by the two NK cell sub-populations.

Blocking KCa3.1 with TRAM-34 induced a depolarization of the plasma membrane of A-NK cells and an increase in their degranulation and cytotoxicity towards K562 target cells. ShK-186 induced the depolarization of the plasma membrane of NA-NK cells but in those cells, the blocker had inhibitory effects on proliferation and degranulation. These findings are consistent with previous work that demonstrated that depolarization did not cause the inhibition of NK cell cytotoxicity induced by Kv channel blockers [43].

The similarity in conjugate formation between non-treated and TRAM-34-treated cells rules out a regulatory role of KCa3.1 in the adhesion of NK cells to K562 cells and suggests that TRAM-34 is not affecting the cell-attachment step of the killing process.

We observed a similar pattern of migration for TRAM-34 treated and non-treated A-NK cells. Furthermore, the migration patterns of TRAM-34-treated NA-NK cells and non-treated-NA-NK cells were also comparable. These results suggest that TRAM-34 does not increase NK cell cytotoxicity towards K562 cells *in vivo* through increased migration to the tumor but rather through increased degranulation.

Mice that received both A-NK cells and TRAM-34 had significantly smaller tumors than mice in the other treatment groups. These data demonstrate a delayed tumor growth and suggest that blocking KCa3.1 with TRAM-34 potentiates the anti-tumor activity of A-NK cells. This enhancing effect of TRAM-34 was not observed in mice treated with NA-NK cells, corroborating our *in vitro* data that showed a preferential effect of TRAM-34 on A-NK cells. Our *in vivo* experiments did not show tumor regression but rather a slowing in tumor growth. This is likely due to the small number of NK cells we infused intravenously (5 million per mouse) combined with the rapid expansion of established tumors. The infusion of 10–30 million human NK cells is necessary to induce subcutaneous tumor regression in NOD-SCID mice when injected intravenously [44], [45]. Our *in vitro* data suggest that the potentiation of the anti-tumor activity of A-NK cells by TRAM-34 is due to an increase in degranulation and cytotoxicity. Although the expression of chemokine receptors was not affected by TRAM-34 in A-NK cells, it is still possible that TRAM-34 may alter the homing potential of these cells; this will warrant further studies.

KCa3.1 is expressed on a number of primary tumor cells, including glioblastoma and melanoma; blocking those channels reduces the invasiveness and proliferation of those tumors and induces their apoptosis [46]–[48]. Since NK cells play an important role in killing tumor cells, targeting KCa3.1 with selective blockers may prove beneficial in cancer therapy by affecting both the NK cells and the tumor cells.

Some tumor cells produce MMP-23, a large protein that contains a domain analogous to the Kv1.3 blocker ShK [49]. This may represent a mechanism for tumor cells to reduce their destruction by immune cells. Our data suggest that A-NK cells will remain effective in proliferating and killing such tumor cells through their reliance on KCa3.1 rather than Kv1.3 channels.

Understanding the mechanisms by which NK cells regulate their proliferation and the release of cytotoxic granules to kill target cells is important in NK cell therapy against diseased cells. The precise mechanisms by which KCa3.1 regulates degranulation by A-NK cells warrants further investigation, as do the mechanisms regulating expression of Kv1.3 and KCa3.1 by the different subsets of human NK cells.

## Supporting Information

Figure S1 **Representative flow cytometric histograms comparing surface expression levels of CD3, CD56, CD16, CD11b, and CD27 by freshly isolated NK cells (unseparated cells), and by A-NK and NA-NK cells after overnight incubation with rhIL-2 and rhIL-15, separation and incubation in cytokine-free medium for 24 hours.** Quantification of the different subpopulations is shown on the right for each marker.
(TIF)

Figure S2 **Representative flow cytometric histograms comparing surface expression levels of CCR7, CD62L, CD69, CD90, and CD134 by freshly isolated NK cells (unseparated cells), and by A-NK and NA-NK cells after overnight incubation with rhIL-2 and rhIL-15, separation and incubation in cytokine-free medium for 24 hours.** Quantification of the different subpopulations is shown on the right for each marker.
(TIF)

Methods S1 **Flow cytometric methods and reagents used in supporting figures S1 and S2.**
(DOC)
