# Supplementary figures and images for: Blocking KCa3.1 Channels Increases Tumor Cell Killing by a Subpopulation of Human Natural Killer Lymphocytes
Source: PLoS One. 2013 Oct 11;8(10):e76740. doi: 10.1371/journal.pone.0076740 (PMC3795664; doi:10.1371/journal.pone.0076740)

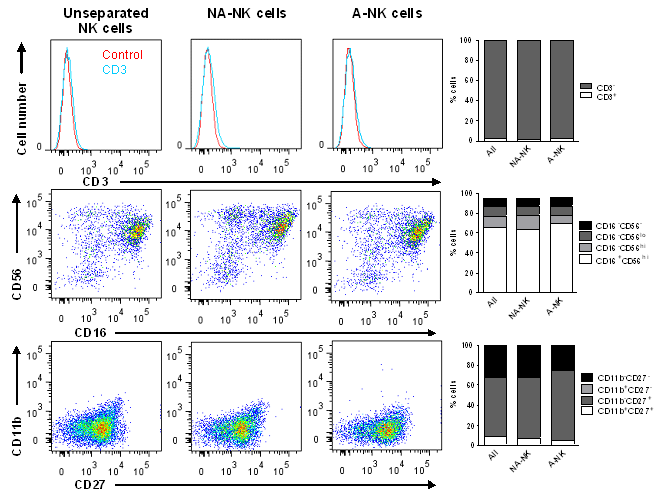

Supplement: Figure S1 — Representative flow cytometric histograms comparing surface expression levels of CD3, CD56, CD16, CD11b, and CD27 by freshly isolated NK cells (unseparated cells), and by A-NK and NA-NK cells after overnight incubation with rhIL-2 and rhIL-15, separation and incubation in cytokine-free medium for 24 hours. Quantification of the different subpopulations is shown on the right for each marker. (TIF) [file pone.0076740.s001.tif]

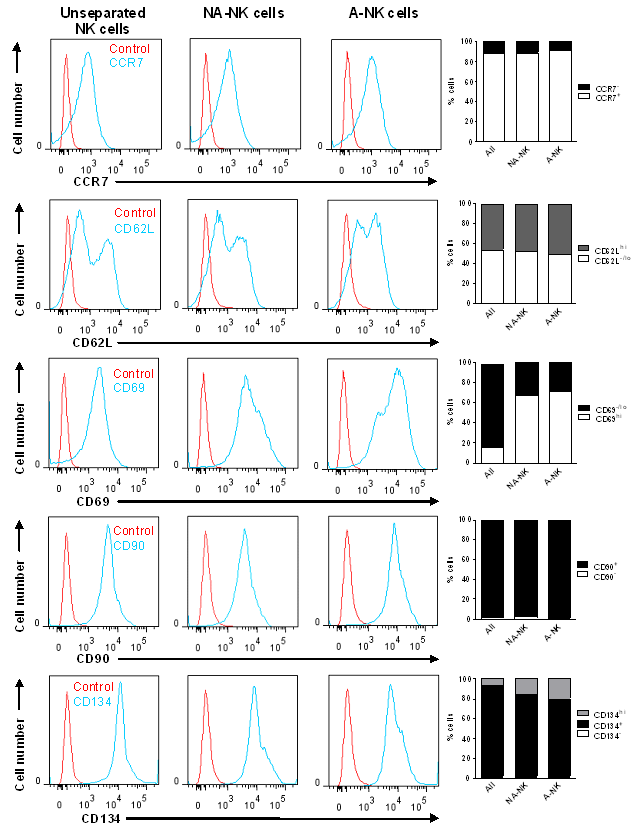

Supplement: Figure S2 — Representative flow cytometric histograms comparing surface expression levels of CCR7, CD62L, CD69, CD90, and CD134 by freshly isolated NK cells (unseparated cells), and by A-NK and NA-NK cells after overnight incubation with rhIL-2 and rhIL-15, separation and incubation in cytokine-free medium for 24 hours. Quantification of the different subpopulations is shown on the right for each marker. (TIF) [file pone.0076740.s002.tif]
