# Supplementary material for: Blocking KCa3.1 Channels Increases Tumor Cell Killing by a Subpopulation of Human Natural Killer Lymphocytes
Source: PLoS One. 2013 Oct 11;8(10):e76740. doi: 10.1371/journal.pone.0076740 (PMC3795664; doi:10.1371/journal.pone.0076740)
Supplement: Methods S1 — Flow cytometric methods and reagents used in supporting figures S1 and S2. (DOC) [file pone.0076740.s003.doc]

**Supplemental methods - flow cytometry.** NK cells (0.1 - 0.5 million per tube) were washed with FACS-PBS (PBS + 2% goat serum + 2% BSA + 1% human AB serum) and incubated for 20 min on ice in the dark with fluorophore-conjugated antibodies at concentrations recommended by the manufacturer. Cells were thoroughly washed, fixed with paraformaldehyde (1% in PBS), and data acquired on a BD Canto II flow cytometer with FACSDiva (BD Biosciences) and analyzed with FlowJo (Treestar, Ashland, OR) softwares. Antibodies were purchased from BD Biosciences (anti-CD3 conjugated to PE, anti-CD16 conjugated to FITC, anti-CD11b conjugated to APC, anti-CD27 conjugated to FITC, anti-CCR7 conjugated to Alexa Fluor 647, anti-CD29 conjugated to APC, anti-CD90 conjugated to FITC, anti-CD25 conjugated to FITC, isotype controls), eBiosciences (anti-CD134 conjugated to FITC, anti-CD62L conjugated to FITC, anti-CD69 conjugated to Alexa Fluor 647), or R&D Systems (anti-CD56 conjugated to APC).
